# Dihydroartemisinin Induces Ferroptosis in HCC by Promoting the Formation of PEBP1/15-LO

**DOI:** 10.1155/2021/3456725

**Published:** 2021-12-10

**Authors:** Ying Su, Danli Zhao, Chun Jin, Zhanghao Li, Sumin Sun, Siwei Xia, Yuxin Zhang, Zili Zhang, Feng Zhang, Xuefen Xu, Jiangjuan Shao, Biyun Zhang, Shizhong Zheng

**Affiliations:** ^1^Jiangsu Key Laboratory for Pharmacology and Safety Evaluation of Chinese Materia Medica, Nanjing University of Chinese Medicine, Nanjing, China; ^2^Jiangsu Health Vocational College, Nanjing, China; ^3^Department of Nuclear Medicine, Jiangsu Province Hospital of Chinese Medicine, Affiliated Hospital of Nanjing University of Chinese Medicine, China

## Abstract

Relevant researches have recognized the vital role of inducing ferroptosis in the treatment of tumor. The latest findings indicate that PEBP1/15-LO can play an essential role in the process of cell death. However, its role in regulating ferroptosis in hepatocellular carcinoma (simplified by HCC) remains unclear. The previous research of our team has proved that DHA can induce ferroptosis of hepatic stellate cells. In this study, we found that DHA could also induce ferroptosis in HCC cells. Interestingly, DHA induced ferroptosis by promoting the formation of PEBP1/15-LO and promoting cell membrane lipid peroxidation. In addition, we also found that DHA had no obvious regulatory effect on 15-LO, but it could promote PEBP1 protein expression. Importantly, we discovered the upregulation of PEBP1 induced by DHA was related to the inhibition of its ubiquitination degradation. In vivo experiments have also obtained consistent results that DHA can inhibit tumor growth and affect the expression of ferroptosis markers in tumor tissues, which would be partially offset by interference with PEBP1.

## 1. Introduction

Hepatocellular carcinoma (HCC) is the most common type of liver cancer, with an extensive global influence. The global incidence is increasing year by year, and the mortality rate has ranked third in tumor-related deaths [1]. HCC is found usually in the middle and advanced stage and has been unable to carry out effective surgical treatment. Therefore, HCC is a serious threat to human health and life. New and effective treatment regimens are urgently needed. For a long time, the primary drugs used in tumor treatment are chemical synthesis drugs, which are extensively used but all have a number of serious side effects [2]. Hence, it is urgent to find antitumor drugs that can selectively kill tumor cells without obvious toxicity to normal cells. Traditional Chinese medicine effective component can target and regulate the expression of some proteins and genes of cell function, which makes the traditional Chinese medicine monomer compounds in the treatment of HCC play a more and more important role. Therefore, it is particularly important to find effective traditional Chinese medicine monomers for the prevention and treatment of liver cancer.

Ferroptosis was discovered by Dixon and Stockwell when studying the mechanism of small molecule erastin killing tumor cells containing oncogene RAS mutation [3]. The main mechanism of ferroptosis is to catalyze the liposome peroxidation of unsaturated fatty acids highly expressed on the cell membrane under the action of divalent iron or lipoxygenase, thereby inducing cell death. A large number of researches have shown that ferroptosis inducers play a critical role in inhibiting HCC growth and killing HCC cells. Sorafenib is the first drug to systematically treat advanced HCC and significantly prolong survival in patients with HCC. In HCC cell lines, the toxicity of sorafenib is significantly reduced after treatment with the iron-chelating agent deferoxamine (DFX), and this inhibition is reversed by lipophilic antioxidants [4, 5]. Therefore, ferroptosis may become a new treatment strategy for HCC.

It has been confirmed that cell death caused by ferroptosis arises from lipid peroxidation [6, 7]. In this chemical reaction, oxygen is added to the polyunsaturated tails of phospholipids in cell membranes, which produces a novel molecule known as lipid hydrogen peroxide and other derivative species that may interfere with cellular lipid membranes' assembly and structure [8]. In some cases, this process can be catalyzed from a class of enzymes called lipoxygenases (LOXs) [9, 10].

Lipoxygenases (LOXs) are nonheme, iron-containing enzymatic protein effectors and an important enzyme system that mediates the formation of ferroptosis peroxides. Free polyunsaturated fatty acids are preferred substrates for LOXs, and LOX knockout can alleviate erastin-induced ferroptosis damage [11]. LOXs involved in mediating lipid peroxides are mainly 15-LO. Recently, it has been found that 15-LO oxidizes free polyunsaturated fatty acids with the help of phosphatidylethanolamine-binding protein (PEBP1) to form a PEBP1/15-LO complex [12, 13]. The allosteric regulation of LOXs can then initiate the downstream phospholipase A2-related oxidation pathway of specific polyunsaturated fatty acids. 15-LO is a novel partner of PEBP1, and the complex formed by the two can allosterically activate LOXs to initiate the ferroptosis process.

Dihydroartemisinin (DHA), a derivative of artemisinin, plays a crucial role in antiviral and antibacterial activities in addition to its antimalarial activity [14]. In addition, the antitumor effect of DHA has also become a focus of research. At present, a large number of studies have shown that DHA has inhibitory effects on lung cancer, prostate cancer, and epithelial ovarian cancer [15–17]. The mechanism research is mainly concentrated in DHA-induced autophagy, cell cycle blocking, and apoptosis, which in turn leads to restricted tumor cell proliferation. Previous researches have confirmed that DHA can also result in iron homeostasis imbalance in cancer cells, which in turn leads to ferroptosis [18]. In addition, previous studies by our team have confirmed that DHA can induce ferroptosis of hepatic stellate cells and reverse the development of hepatic fibrosis [19]. Here, we will discuss whether DHA can induce HCC ferroptosis and exert an antitumor effect by regulating the formation of the PEBP1/15-LO complex.

## 2. Materials and Methods

### 2.1. Antibodies and Reagents

Antibodies against Ki67 (A11005), GPX4 (A11243), SLC7A11 (A2413), and *β*-actin (AC026) were purchased from Abclonal (Wuhan, China); PTGS2 (66351-1-lg) was purchased from Proteintech (Rosemont, IL, USA). Antibodies to PEBP1 (sc-376925) and 15-LO (sc-133085) were bought from Santa Cruz Biotechnology (Dallas, TX, USA). Anti-ubiquitin antibody (#ab7780) was purchased from Abcam Technology (Abcam). DHA (D7439), ferrostatin-1 (Ferr-1, SML0583), dimethylsulfoxide (DMSO), chloroquine (CQ), and cycloheximide (CHX) were supplied by Merck KGaA (Darmstadt, Germany). Dulbecco's modified eagle medium (DMEM), fetal bovine serum (FBS), phosphate-buffered saline (PBS), and Opti-MEM medium were bought from GIBCO BRL (Grand Island, NY).

### 2.2. Cell Culture

Human hepatoma cell lines Huh-7 and HepG2 were purchased from the Cell Bank of Chinese Academy of Sciences (Shanghai, China). And the cells were cultured in DMEM with 10% FBS and 1% antibiotics. PEBP1 overexpression plasmid, 15-LO siRNA, and PEBP1 siRNA were synthesized by KeyGEN Biotechnology Co., Ltd. (Nanjing, China). Mix the 15-LO siRNA, PEBP1 siRNA or PEBP1 overexpression plasmid, HiFectPlus Transfection Reagent, and HiFectPlus Enhanced Diluent at a ratio of 1 : 2 : 100 according to the Thikarrow HiFectPlus Transfection Reagent instruction manual, and then, complete the preparation of the transfection working solution. Leave it at indoor temperature for 20-30 minutes and then evenly drop it into the culture dish incubated with cells (the ratio of transfection working solution to complete medium is about 1 : 10). The cell transfection efficiency was verified by western blot analysis.

### 2.3. RNA Isolation and Real-Time PCR Analyses

Total RNA was isolated from HCC cells or tissues using TRIzol™ Reagent (Sigma, Saint Louis, MO, USA) and reverse transcribed to cDNA using the First Strand cDNA Synthesis SuperMix for qPCR (gDNA digester plus) Kits from Yeasen Biotech Co., Ltd. (Shanghai, China). Fast SYBR™ Green Master Mix (Yeasen Biotech Co., Ltd. Biotech Co., Ltd.) was used to perform real-time PCR analysis. The amounts of transcript were normalized to those for GAPDH. The Primer (Tsingke Biotechnology Co., Ltd., Nanjing, China) is presented in Table 1.

### 2.4. Western Blot Analyses

RIPA lysis buffer (Sigma-Aldrich, R0278) with protease inhibitors (1%) and phosphatase inhibitors (1%) was utilized to lyse the cells or tumors for 30 min, and lysates were harvested by centrifugation (12,000 rpm) at 4°C for 10 min. The extracted protein was detected and quantified using the Pierce™ BCA Protein Assay Kit (23250, Thermo Scientific) and referring to the experimental method described previously [20] for the next western blot analysis. *β*-Actin was used as the invariant control for total protein.

### 2.5. Cell Proliferation Assay

The cell proliferation assay was determined by MTT (Biosharp, Nanjing, China). Briefly, 2 × 10^4^ HepG2 or Huh-7 cells were plated into 96-well plates per well. After treatment with specific concentrations of DHA or Ferr-1 for 24 h, the cells were incubated with 20 *μ*L MTT solution (5 mg/mL) at 37°C for 4 h. Then, discard the culture medium and use 200 *μ*L DMSO solution for each well to dissolve the bottom crystals. The absorbance values of each well were detected by using a SpectraMax™ microplate spectrophotometer (Molecular Devices, Sunnyvale, CA) at 490 nm. EdU cell proliferation was performed using EDU kit (BeyoClick™ EdU Cell Proliferation Kit with Alexa Fluor 488, Beyotime, China). Simply put HepG2 or Huh-7 cells were inoculated into 6-well plates and treated with drugs as required after cell adherence. Then, it was incubated with EdU for 3 h, fixed with 4% paraformaldehyde for 15 min, and infiltrated with 0.3% Triton X-100 for 10 min. Incubate with Click Reaction Mixture at room temperature for 30 min away from light, and then, detect with fluorescent microplate detector.

### 2.6. Cytotoxicity Test

The toxicity test of DHA was conducted by lactate dehydrogenase (LDH) cytotoxicity kit, and the specific experimental method was performed referring to the protocol in the LDH kit (Beyotime Biotechnology; # C1007).

### 2.7. Iron, GSH, MDA, and 4-HNE Measurement

The Iron Assay kit (# ab83366) was used to detect iron in HCC cells. MDA detection kit (Best Bio; # BB4709) was used to measure the amount of MDA in cells. 4-HNE ELISA kit (YIFEIXUE BIOTECH; # YFXEH00631) was used to measure intracellular 4-HNE. Glutathione kit (Best Bio; # BB-4711) was used to estimate the GSH content in HCC cells. All experimental methods were performed with reference to the protocols in the corresponding kit.

### 2.8. ROS Measurement

After the sterile slides were laid in the 24-well plates, about 8 × 10^4^ HCC cells were planted in each well. After the cells adhered to the wall, treated with different concentrations of DHA, followed by DCFH-DA (Sciben Biotech Co., Ltd.; #MR1008) which was diluted into 10 mmol/L with DMEM, cells were placed in an incubator at 37°C with DMEM containing 10 mmol/L DCFH-DA probe for 20 min. Remove the medium containing DCFH-DA and wash three times with serum-free medium to completely remove the probes outside the cell. The cover glass was then removed, and the representative image was observed and taken a picture using an inverted Zeiss fluorescence microscope (Axio Observer A1).

### 2.9. Immunoprecipitation Experiment

After treatment with 20 *μ*M DHA for 24 h, the cells were collected and lysed at 4°C with RIPA buffer containing protease inhibitor MG132. The antigen-containing lysate supernatant was added to the target antibody and placed on the turnover mixer for overnight reaction at 4°C. And then, the antigen-antibody binding complex was mixed with the Protein A/G Plus MagPoly Beads (Abclonal Technology; #RM09008) at 4°C overnight. After that, the supernatant was centrifuged and collected, and the precipitation was washed twice with buffer solution (20 mM Na_2_HPO_4_, 0.15 M NaCl, and pH 7.0). Whereafter, 30 *μ*L 2× SDS-PAGE Loading Buffer was added and mixed well and heated at 95°C for 15 min. The supernatant was collected by centrifugation, and the proteins were isolated by SDS-PAGE and analyzed by western blot.

### 2.10. Immunofluorescence Staining

Immunofluorescence staining was utilized to evaluate protein expression in HCC tissues and cells. The immunofluorescence staining was carried out according to our previous instructions [21]. After staining the nucleus with DAPI (Sigma; # D9542), three randomly selected fields of view were photographed under laser confocal microscopy (Leica Microsystems AG).

### 2.11. Ubiquitination Analysis

The experimental method of ubiquitination referred to our previously published articles and was slightly improved [22]. Transfected with PEBP1 overexpression plasmid for 12 h, the cells were treated with drugs as needed for another 24 h. Then, 20 *μ*M MG132 was added 6 h before cells collection to prevent protein degradation. After washing with PBS three times, the cells were lysed with 150 *μ*L RIPA buffer, which containing 10 mM N-ethylmaleimide (NEM) (Sigma-Aldrich, E3876). Then, the cells were scraped, collected, lysed on ice for 30 min, and centrifuged at 4°C at 12,000 rpm for 15 min. The supernatant was mixed with an anti-PEBP1 antibody and Protein A/G Plus MagPoly Beads in the inversion mixer at 4°C to react for 2 h or overnight. The supernatant was collected by centrifugation for subsequent detection. After the precipitation was sufficiently washed, 30 *μ*L 2× SDS-PAGE Loading Buffer was added and mixed well and heated at 95°C for 15 min and then separated by SDS-PAGE. Anti-ubiquitin antibody (Abcam Technology, AB7780) was used to detect ubiquitin protein by western blot.

### 2.12. Animal Treatment

Male nude mice (BALB/C-nu/nu) were purchased from the Nanjing Institute of Biomedicine (Nanjing, China). After the nude mice were adaptively fed for one week, 2 × 10^7^ Huh-7 cells were subcutaneously injected into the left ventricle of nude mice to establish the subcutaneous xenograft model. As previously described [23], DHA was dissolved in 10% DMSO and 5% Kolliphor HS 15 in PBS and injected intraperitoneally once daily for about 3 weeks when the tumor grew to 150 mm^3^. All the mice were divided into six groups (four or five animals per group), including model group, DHA low, medium- and high-dose group [19], PEBP1 interference group, and DHA administration with PEBP1 interference group. Mice were anaesthetized by injecting pentobarbital (50 mg/kg) and then sacrificed at the end of the experiment. Liver and tumor tissues were fixed in 10% formalin for histopathological studies. The experimental scheme was approved by the Animal Welfare Institute of Nanjing University of Chinese Medicine (Nanjing, China). According to the National Institutes of Health guidelines, all animals are cared for humanely.

### 2.13. Tumorigenesis Assays in Nude Mice

The long (a) and wide (b) sides of the tumor in nude mice were measured with a vernier caliper every two days. Tumor volume was estimated using the following formula, tumor volume = (*a* × *b*^2^)/2.

### 2.14. Statistical Analysis

Data from at least triplicate experiments were expressed by mean ± standard deviation. All statistical analyses between groups were performed by either one-way ANOVA followed by Tukey's multiple comparison test or unpaired Student's *t*-test, using GraphPad Prism 8.0 (San Diego, CA, USA). Data significance is expressed as significant (^∗^*p* < 0.05 or ^#^*p* < 0.05), very significant (^∗∗^*p* < 0.01 or ^##^*p* < 0.01), and highly significant (^∗∗∗^*p* < 0.001 or ^###^*p* < 0.001).

## 3. Results

### 3.1. DHA Inhibits HCC Development In Vitro and Vivo

**Figure 1:**
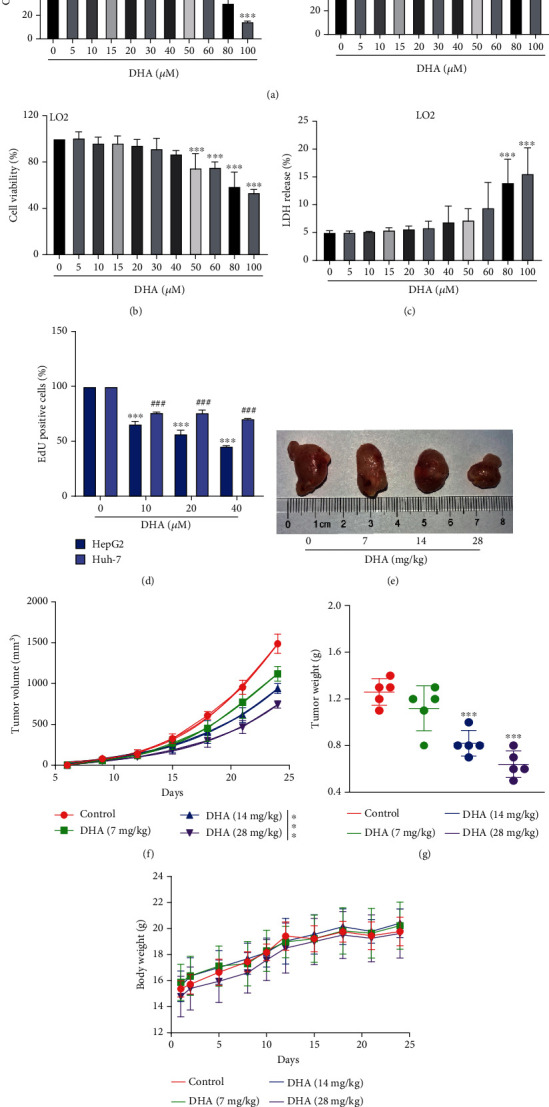
DHA inhibits HCC development in vitro and vivo. (a, b) Cell viability was tested through MTT experiment. Different dosages of DHA were added to the culture medium of HepG2, Huh-7, and LO2 cells. (c) Cell toxicity was detected by LDH release assay. LO2 cells were dealt with varying doses of DHA. (d) Cell proliferation assay was performed with EdU kit. Model mice were divided into the vehicle control and DHA (7, 14, and 28 mg/kg) groups (five mice in every group) at random. (e) Representative photos of tumors stripped from mice at day 24. The (f) tumor volume and (h) body weight of mice in each group during the whole experiment period. (g) Tumor weight was obtained after executing mice on the last day (*n* ≥ 5, ^∗^*p* < 0.05, ^∗∗^*p* < 0.01, and ^∗∗∗^*p* < 0.001).

To clarify the effect of DHA in the development of HCC cells, HepG2 and Huh-7 cells were intervened with gradient DHA (0-100 *μ*M) for 24 h, respectively, and then, cell proliferation was detected by MTT assay. As shown in Figure 1(a), DHA significantly reduced the viability of HepG2 and Huh-7 cells in a dose-dependent manner compared with the control group. Surprisingly, DHA had no significant effect on human normal liver cell line LO2 viability before 50 *μ*M (Figure 1(b)). Similarly, the cell death ratio, which was tested by the LDH release assay, showed that the concentration of DHA could only produce toxicity to LO2 above 80 *μ*M (Figure 1(c)). Therefore, three effective concentrations of DHA acting on HCC, 10, 20, and 40 *μ*M, respectively, were selected for subsequent experiments without having any toxic effect on normal liver cells. In addition, the effect of DHA on HCC cell proliferation was further verified by EdU incorporation experiment, and the results were consistent (Figure 1(d)).

To investigate the antitumor effect of DHA in vivo, Huh-7 cells were inoculated subcutaneously in nude mice to establish subcutaneously transplanted tumor model. As shown in Figures 1(e)–1(g) and Supplementary Fig. 1(a), compared with model control mice, DHA treatment significantly inhibited tumor growth. In addition, the drug had little effect on the bodyweight of mice, indicating that DHA had no obvious side effects on mice at this concentration (Figure 1(h)). In summary, DHA inhibited HCC development in a dose-dependent manner both in vitro and vivo.

### 3.2. DHA Facilitates HCC Cell Ferroptosis In Vitro

**Figure 2:**
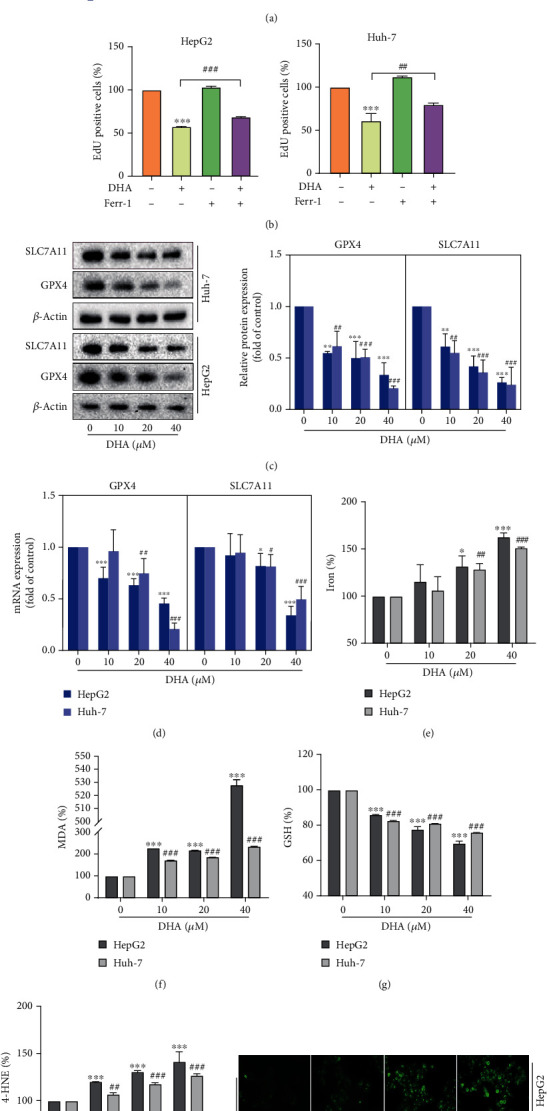
DHA facilitated HCC cell ferroptosis in vitro. HCC cells were treated with the prescribed concentration of DHA for 24 h. (a) The effects of different concentrations of DHA on the viability of HepG2 and Huh-7 cells were determined by MTT assay in the presence or absence of ferroptosis inhibitor Ferr-1 (10 *μ*M). (b) Cell proliferation assay was performed with EdU kit. HepG2 and Huh-7 cells treated with DHA (20 *μ*M) or Ferr-1 (10 *μ*M). (c) The protein levels of SLC7A11 and GPX4 were quantitatively analyzed by western blot, and their quantitative maps were drawn. (d) Real-time PCR were applied to detect the mRNA levels of ferroptosis markers SLC7A11 and GPX4. The levels of (e) iron, (f) MDA, and (g) GSH in HCC cells were tested via using corresponding kits. (h) The level of 4-HNE in HepG2 and Huh-7 cells was tested via ELISA assay. (i) ROS probe was used to detect the level of ROS in HCC cells (*n* ≥ 3, ^∗^*p* < 0.05, ^∗∗^*p* < 0.01, ^∗∗∗^*p* < 0.001, ^#^*p* < 0.05, ^##^*p* < 0.01, and ^###^*p* < 0.001).

Studies have shown that artemisinin and its derivatives can effectively induce tumor cell ferroptosis [24, 25]. In addition, DHA was recently shown to induce ferroptosis in hepatic stellate cells [19]. Therefore, we hypothesized that DHA exerted antitumor activity in this study mainly by regulating HCC ferroptosis. To verify this conjecture, firstly, we used a ferroptosis inhibitor Ferr-1 for preliminary validation. The results of MTT assay showed that the addition of Ferr-1 significantly alleviated the inhibitory effect of DHA on HCC cell proliferation (Figure 2(a)). And the results of the EdU experiment were also consistent (Figure 2(b)). These results suggested that DHA exacted its tumor-inhibiting effect in part by inducing ferroptosis in HCC. Next, we examined the changes of ferroptosis indexes in HCC cells after DHA treatment.

We were pleasantly surprised to observe the downregulation of ferroptosis markers glutathione peroxidase 4 (GPX4) and cystine/glutamate antiporter SLC7A11 at both protein and mRNA levels in a dose-dependent manner after DHA treatment (Figures 2(c) and 2(d)). Then, as we expected, the contents of iron and lipid peroxidation products MDA and 4-HNE all increased to varying degrees, except for reduced GSH (Figures 2(e)–2(h)). In addition, the accumulation of intracellular ROS is an important cause and a major measurement index of ferroptosis. Therefore, ROS fluorescence probe was used to test the changes of ROS level in HCC cells after DHA treatment, and the results showed that the fluorescence intensity increased with the increase of DHA concentration (Figure 2(i)), indicating that DHA could induce the accumulation of ROS in HCC cells. Overall, our data showed that DHA induced ferroptosis in HCC cells.

### 3.3. DHA Induces HCC Cells Ferroptosis by Promoting the Formation of PEBP1/15-LO Complex

**Figure 3:**
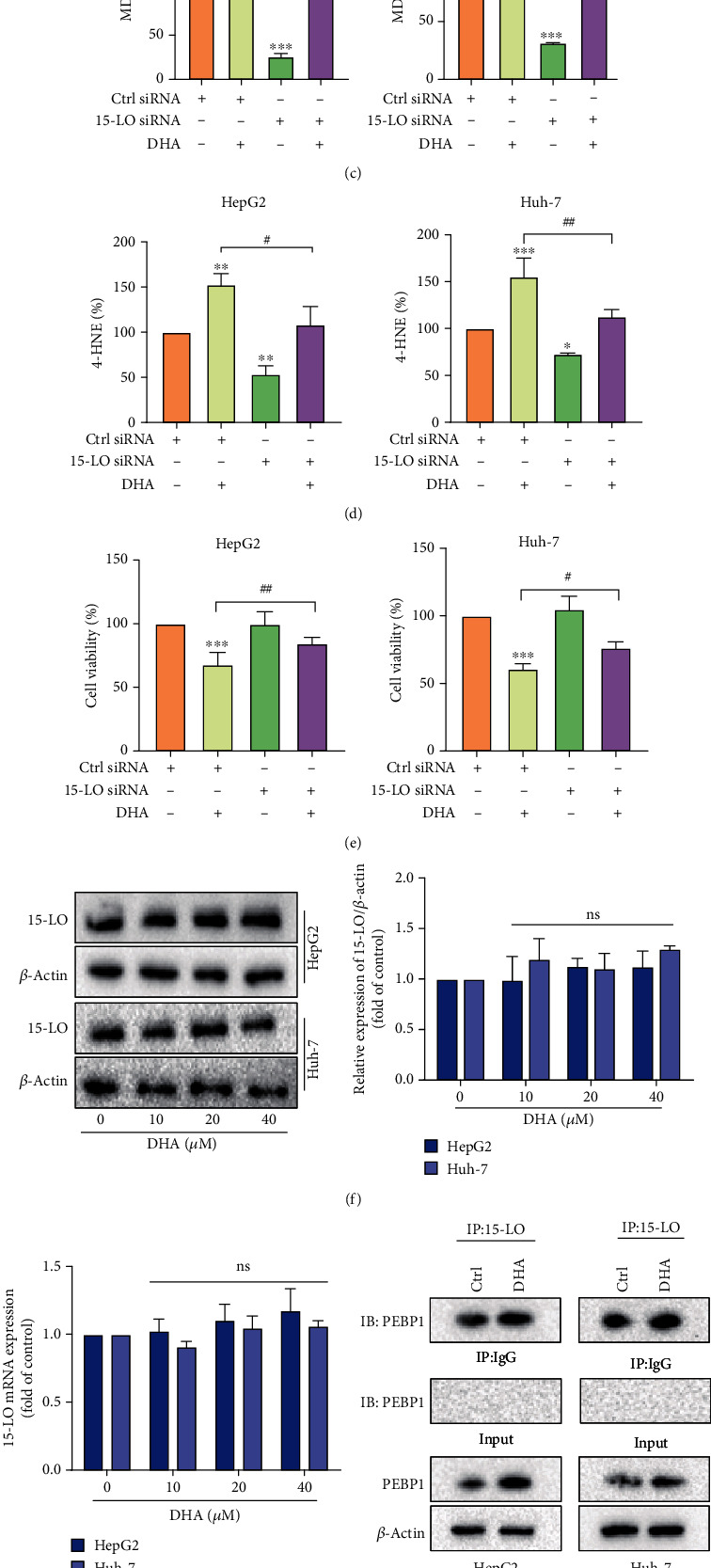
DHA induced HCC cell ferroptosis by promoting the formation of PEBP1/15-LO complex. HepG2 cells and Huh-7 cells were transfected with 15-LO siRNA for 12 h and then treated with different concentrations of DHA for 24 h. (a) The transfection efficiency of si15-LO in HepG2 and Huh-7 cells was detected by western blot. (b, c) The levels of reductive GSH and lipid peroxide MDA were analyzed by the corresponding kits. (d) The level of 4-HNE in HCC cells was determined by ELISA kit. (e) HCC cell viability was detected with MTT kit. (f, g) Western blot and real-time PCR analyses of 15-LO expression in HCC cells treated with DHA (0, 10, 20, and 40 *μ*M) for 24 h. HCC cells were treated with or without DHA (20 *μ*M) for 24 h. (h) The proteins extractive from HCC cells were coincubated with 15-LO antibody and Protein A/G Plus MagPoly Beads. Western blot was used to detect the interactions between PEBP1 and 15-LO. (i) HCC cells were fixed in 1% BSA and incubated with 15-LO and PEBP1 antibodies overnight at 4°C. The colocalization of PEBP1 and 15-LO was detected by double immunofluorescence staining (*n* ≥ 3, ^∗^*p* < 0.05, ^∗∗^*p* < 0.01, ^∗∗∗^*p* < 0.001, ^#^*p* < 0.05, ^##^*p* < 0.01, and ^###^*p* < 0.001).

Next, we sought to explore the potential molecular mechanisms of DHA-induced HCC cell ferroptosis. We have previously determined that cell death by ferroptosis is a result of lipid peroxidation, and the major LOX involved in mediating lipid peroxides is 15-LO [26, 27]. Firstly, we speculated that 15-LO played an important role in inducing HCC cell ferroptosis by DHA. To test this conjecture, 15-LO siRNA was used to pretreat HCC cells and the interference efficiency was verified by western blot (Figure 3(a)) and then conducted subsequent experiments. The results showed that si15-LO treatment canceled the role of DHA in inducing HCC cell ferroptosis, which were confirmed after analyzing of GSH, MDA, and 4-HNE (Figures 3(b)–3(d)). In addition, si15-LO significantly inhibited DHA-induced decline in HCC cell viability (Figure 3(e)). The above data suggested that DHA-induced ferroptosis in HCC cells required the involvement of 15-LO.

However, we were amazed to discover that DHA could not alter the expression of 15-LO at either protein or mRNA level in HCC cells (Figures 3(f) and 3(g)). So how was 15-LO involved in mediating this death effect? Recently, Wenzel et al. [12] found that PEBP1 could accidentally bind to lipoxygenase 15-LO, thereby altering its substrate specificity, promoting membrane lipid peroxidation, and inducing ferroptosis.

We speculated whether the formation of PEBP1/15-LO complex was a prerequisite for DHA to induce ferroptosis in HCC cells. So, we next carried out coimmunoprecipitation to validate whether PEBP1 interacted with 15-LO and the effect of DHA treatment on the interaction between PEBP1 and 15-LO. In both HepG2 and Huh-7 cells, PEBP1 was detectable in the precipitant mediated by the specific antibody of 15-LO (Figure 3(h)). Furthermore, we observed the colocalization of PEBP1 and 15-LO in the intracellular by confocal laser microscopy (Figure 3(i)). And DHA treatment significantly enhanced this interaction and colocalization (Figures 3(h) and 3(i)). These results indicated that DHA could induce ferroptosis in HCC cells by promoting the formation of PEBP1/15-LO complex.

### 3.4. Interfering with PEBP1 Attenuates Ferroptosis Induced by DHA in HCC Cells

**Figure 4:**
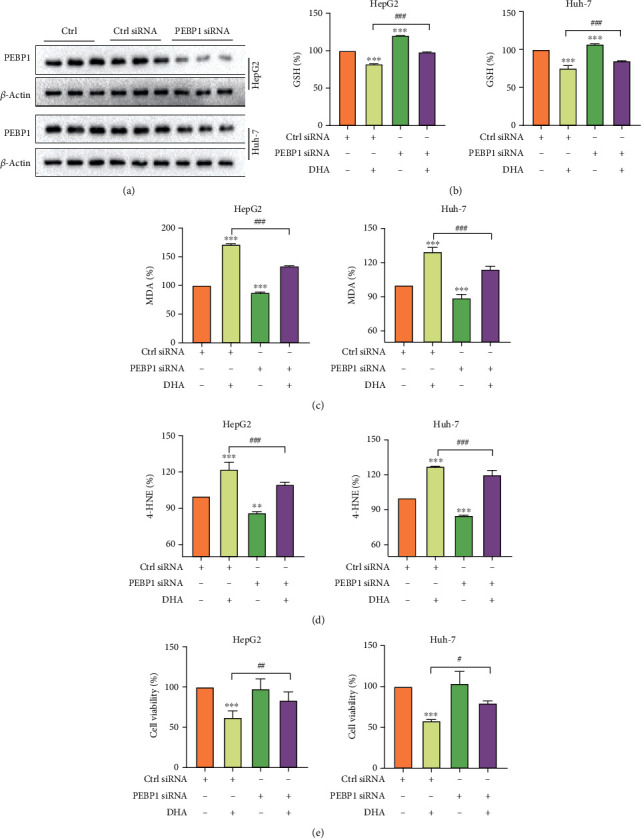
Interfering with PEBP1 attenuated ferroptosis induced by DHA in HCC cells. HepG2 cells and Huh-7 cells were transfected with PEBP1 siRNA for 12 h and then treated with different concentrations of DHA for 24 h. (a) Western blot was used to detect the transfection result of siPEBP1 in HepG2 and Huh-7 cells. (b–d) The levels of reductive GSH, lipid peroxide MDA, and 4-HNE were analyzed by the corresponding kits. (e) The MTT kit was used to assess the viability of HCC cells (*n* ≥ 3, ^∗^*p* < 0.05, ^∗∗^*p* < 0.01, ^∗∗∗^*p* < 0.001, ^#^*p* < 0.05, ^##^*p* < 0.01, and ^###^*p* < 0.001).

The previous research results proved the role of 15-LO in DHA-induced ferroptosis, and DHA could significantly promote the combination of PEBP1 and 15-LO. Next, we want to explore whether PEBP1 is necessary for DHA to induce HCC ferroptosis. Consistently, HepG2 and Huh-7 cells were first treated with PEBP1 siRNA. The transfection efficiency of PEBP1 was verified by western blot analysis (Figure 4(a)). Just as we predicted, we observed that transfection with PEBP1 siRNA could significantly rescue downregulation of GSH and cell viability and inhibited the upregulation of MDA and 4-HNE levels caused by DHA in HCC cells (Figures 4(b)–4(e)), indicating the participation of PEBP1 in the current context.

### 3.5. DHA Promotes the Accumulation of PEBP1 by Inhibiting the Ubiquitination of PEBP1

**Figure 5:**
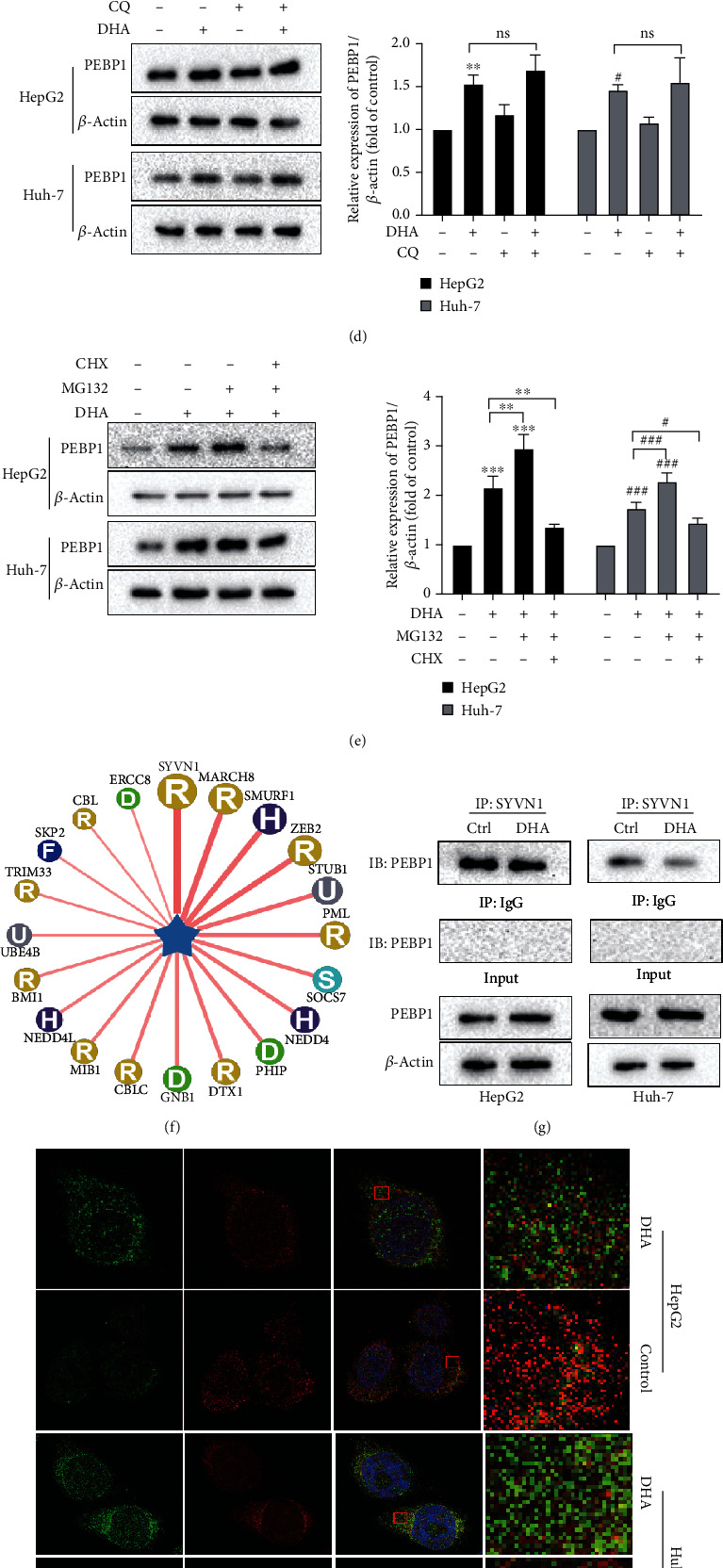
DHA promotes the accumulation of PEBP1 by inhibiting the ubiquitination of PEBP1. The mRNA and protein levels of PEBP1 in HepG2 and Huh-7 cells treated with DHA were evaluated by (a) western blot and (b) real-time PCR. HCC cells were treated with DHA (20 *μ*M) for 24 hours and then added with MG132 (10 *μ*M), CQ (10 *μ*M), or CHX (20 *μ*g/ml) for 6 hours before the collection of cell samples. (c–e) The protein expression of PEBP1 protein was tested by western blot analysis, and its quantitative map was drawn. (f) Predictive diagram of possible E3 ubiquitin ligases interacting with PEBP1. HCC cells were treated with DHA (20 *μ*M) or DMSO for 24 hours. (g) The binding of PEBP1 and SYVN1 was detected by coimmunoprecipitation. (h) The colocalization of PEBP1 and SYVN1 was tested via immunofluorescence. (i) Ubiquitination experiment was used to evaluate the ubiquitination level of PEBP1 (*n* = 3, ^∗^*p* < 0.05, ^∗∗^*p* < 0.01, ^∗∗∗^*p* < 0.001, ^#^*p* < 0.05, ^##^*p* < 0.01, and ^###^*p* < 0.001).

Next, we focused on the regulation of PEBP1 by DHA in HCC cells. We were surprised to find that DHA upregulated the expression of PEBP1 protein but not mRNA in HCC cells (Figures 5(a) and 5(b)). Subsequently, we investigated the effect of DHA on the protein stability of PEBP1. Western blot analysis showed that in the presence of protein synthesis inhibitor CHX, DHA could significantly extend the half-life of PEBP1 protein (Figure 5(c)). Therefore, we hypothesized that DHA had an effect by inhibiting PEBP1 degradation. It is well known that there are two primary approaches to protein degradation, ubiquitin proteasome system and autophagy lysosomal pathway, whereas autophagy inhibitor CQ could not affect the degradation to PEBP1 (Figure 5(d)). Then, we used CHX and the proteasome-specific inhibitor MG132 to explore whether DHA promotes protein accumulation of PEBP1 by inhibiting ubiquitination of PEBP1. As shown in Figure 5(e), CHX was observed to attenuate DHA mediated upregulation of PEBP1, while MG132 further enhanced DHA-mediated PEBP1 overexpression. These results indicated that PEBP1 was degraded through the proteasome pathway. Ubibrowser (http://ubibrowser.ncpsb.org/) is a system that can predict the E3 ubiquitin ligase binding to the target substrate. Through this particular system, we found the E3 ligase most likely to act on PEBP1 was Synoviolin 1 (SYVN1) (Figure 5(f)). Immunoprecipitation results also confirmed the coprecipitation of PEBP1 and SYVN1, and DHA could weaken this binding (Figure 5(g)). Besides, immunofluorescence results also demonstrated the colocalization of PEBP1 and SYVN1, while DHA treatment reduced this colocalization (Figure 5(h)). Ubiquitin experiment further verified that SYVN1 was indeed an ubiquitin ligase of PEBP1, and an obvious reduction of polyubiquitinated PEBP1 protein was observed after DHA treatment in HCC cells (Figure 5(i)). These results suggested that DHA could promote the accumulation of PEBP1 protein by protecting PEBP1 from ubiquitin degradation.

**Figure 6:**
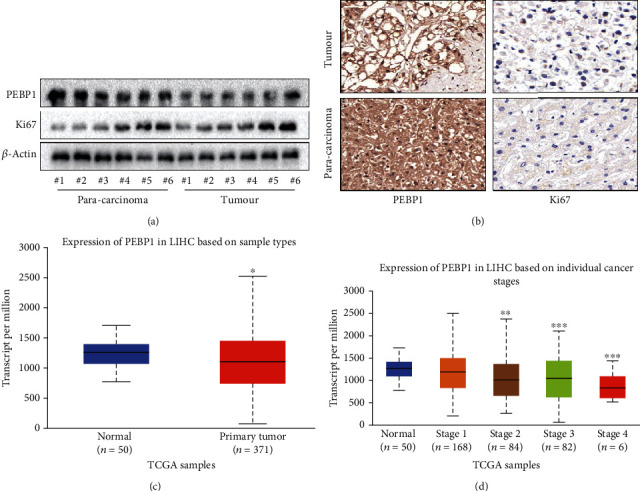
PEBP1 may be a potential prognostic factor for liver cancer. (a) Western blot was used to detect the protein expressions of PEBP1 and Ki67 in HCC tissues and paracarcinoma tissues which were obtained from six HCC patients. (b) Typical images of PEBP1 and Ki67 immunohistochemical staining on HCC and paracarcinoma tissues (scale bar, 50 *μ*m). (c, d) TCGA database showed the expression level of PEBP1 in liver cancer tissues and normal liver tissues and the correlation between the expression of PEBP1 and the malignant degree of liver cancer (*n* ≥ 3, ^∗^*p* < 0.05, ^∗∗^*p* < 0.01, and ^∗∗∗^*p* < 0.001).

Subsequently, we detected and compared the expression of PEBP1, Ki67 in liver cancer, and paracarcinoma tissues. Identical to what have been found [28], the expression of PEBP1 in liver cancer tissues was much lower than that in paracarcinoma tissues. In addition, there was a negative correlation between PEBP1 and Ki67 (Figures 6(a) and 6(b)). The results of TCGA database also showed that PEBP1 was low expressed in liver cancer and negatively correlated with the degree of malignancy in human liver cancer (Figures 6(c) and 6(d)), which further manifested the important role of PEBP1 in the development of HCC.

### 3.6. PEBP1 Is Required for DHA-Mediated Ferroptosis of HCC In Vivo

**Figure 7:**
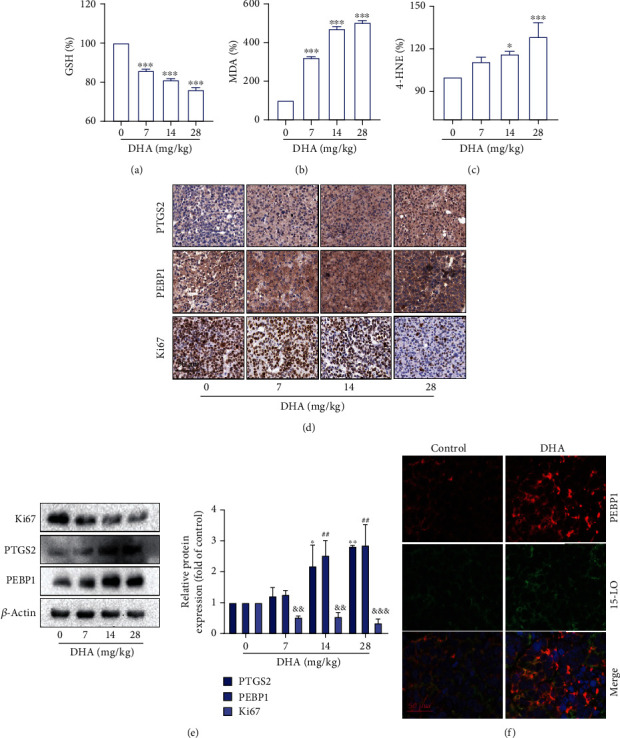
DHA induced ferroptosis of HCC xenograft tumor in vivo. nude mice with Huh-7 cell xenograft. (a, b) The corresponding kits were used for evaluating GSH and MDA levels in tumor tissues. (c) The level of 4-HNE in the tumor tissues was detected by ELISA kit. (d) Immunohistochemical staining and (e) western blot analysis were used to assess PEBP1, PTGS2, and Ki67 levels in tumor tissues, scale bar: 50 *μ*m. (f) Immunofluorescence staining showed the expression levels of PEBP1 and 15-LO and their colocalization in tumor tissues (*n* ≥ 3, ^∗^*p* < 0.05, ^∗∗^*p* < 0.01, ^∗∗∗^*p* < 0.001, ^#^*p* < 0.05, ^##^*p* < 0.01, ^###^*p* < 0.001, ^&^*p* < 0.05, ^&&^*p* < 0.01, and ^&&&^*p* < 0.001).

Then, to validate our results in vitro studies, human hepatoma cell line Huh-7 was used to establish a subcutaneous xenograft model in nude mice. Our previous results have confirmed that DHA has quite a good function on anti-HCC both in vivo and vitro (Figure 1). In this section, we investigated the effects of DHA in ferroptosis and the role of PEBP1 in the xenograft model. Firstly, we examined the changes in ferroptosis-related indicators. That is consistent with our previous cell experiments, compared with the control group, the levels of GSH decreased in DHA-treated mice, while the levels of MDA and 4-HNE increased significantly (Figures 7(a)–7(c)). Furthermore, the results of immunohistochemistry and western blot showed the levels of PEBP1 and ferroptosis marker PTGS2 in the DHA administration group were significantly higher than that in the control group dose-dependently, which were negatively correlated with Ki67 (Figures 7(d) and 7(e)). In addition, DHA treatment could significantly promote the combination of PEBP1 and 15-LO (Figure 7(f)).

**Figure 8:**
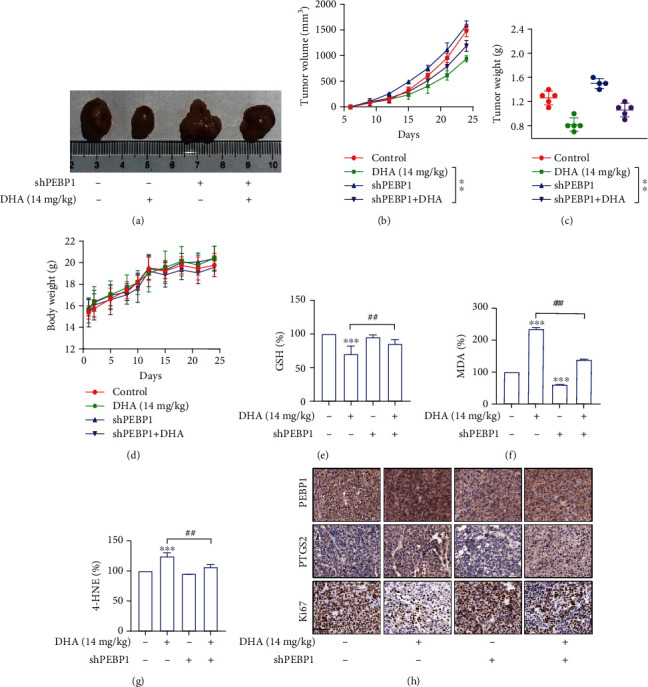
The PEBP1 is a prerequisite for DHA to induce ferroptosis in HCC. Subcutaneous xenografts established by using Huh-7 cells. (a) Representative photos of subcutaneous tumor removed from nude mice on the last day of the experiment. (b) Tumor volume growth curves of mice in each group during the experimental period. (c) The subcutaneous tumors of nude mice were stripped and weighed. (d) Change curve of body weight of mice in the whole experiment cycle. (e, f) The corresponding kit analyses of ferroptosis markers GSH and MDA in the tumor tissue. (g) 4-HNE levels in the tumor tissues analyzed by 4-HNE ELISA kit. (h) Immunohistochemical analyzed the expression of Ki67, PTGS2 and PEBP1 in tumor tissues. Scale bars are 50 *μ*m (*n* = 5, ^∗^*p* < 0.05, ^∗∗^*p* < 0.01, ^∗∗∗^*p* < 0.001, ^#^*p* < 0.05, ^##^*p* < 0.01, and ^###^*p* < 0.001).

Next, we mainly investigated whether the role of DHA in ferroptosis depended on the regulation of PEBP1 in vivo. As seen from the tumor representative map and tumor growth curve, interference with PEBP1 can significantly promote tumor growth. DHA administration group significantly inhibited tumor growth. Interestingly, by interfering with PEBP1, the effect of DHA was weakened (Figures 8(a) and 8(b); Supplementary Fig. 1(b)). At the same time, the changes in tumor weight were consistent when tumor lumps were stripped from each mouse (Figure 8(c)). Furthermore, no marked changes in body weight were observed in mice treated with either DHA or the PEBP1 shRNA, indicating that side effects of DHA and the shRNA were minimal in vivo (Figure 8(d)). The result of ferroptosis-related indexes GSH, MDA, and 4-HNE showed that the effects of DHA were abrogated via interfering with PEBP1 (Figures 8(e)–8(g)). In addition, interference with PEBP1 inhibited DHA-induced upregulation of PTGS2 and downregulation of Ki67 (Figure 8(h)). In short, PEBP1 played an essential role in DHA-induced ferroptosis in the subcutaneous xenograft model.

## 4. Discussion

HCC is a major malignant tumor in the clinical, ranked as the fifth most common diagnosis and the third leading cause of cancer-related death worldwide. Most patients are not suitable for surgical treatment, making drug therapy the preferred option. DHA, a derivative of artemisinin with the C-10 lactone group replaced by hemiacetal, is used to treat various forms of malaria. Recently, it has been found that DHA also has a certain potential in cancer treatment [29–31]. In this study, we confirmed the inhibiting effect of DHA on the development of HCC in vivo and in vitro (Figure 1). Our data showed that DHA could inhibit the viability of HCC cells above 10 *μ*M and would not affect the cell viability of human normal liver cell LO2 below 50 *μ*M. In addition, we also used LDH release experiments to prove that DHA would not be toxic to LO2 cells below 80 *μ*M; therefore, we used 10, 20, and 40 *μ*M DHA for follow-up research. At the same time, in vivo experiments have also effectively proved that DHA could inhibit the development of HCC.

Ferroptosis is a new type of programmed cell death that is different from typical apoptosis and cell necrosis, mainly characterized by elevated intracellular lipid peroxidation and ROS accumulation. Since the concept of ferroptosis came out in 2012, the research on ferroptosis has been increasing year by year. A large number of studies have shown that ferroptosis inducers play an important role in inhibiting the growth of HCC and killing HCC cells. Further investigation into the effects of ferroptosis in cancer will provide a new direction for cancer diagnosis and treatment [32].

DHA has been involved in the treatment of breast cancer, liver cancer, lung cancer, etc. Most studies are regulating cancer cell apoptosis and inhibiting invasion [33–35]. However, DHA-induced ferroptosis in hepatoma cells has not been reported. Our team has previously proved that DHA can induce ferroptosis of hepatic stellate cells [19]. Based on this, this research innovatively proposed that DHA could induce ferroptosis of HCC cells to exert antitumor effects.

Our data are the same as we expected. After DHA treatment, there were significant changes in the ferroptosis-related indicators in HepG2 and Huh-7 cells, such as lipid ROS, lipid peroxide MDA, 4-HNE, and iron accumulation. And DHA also downregulated the expression of GPX4 and SLC7A11 in varying degrees. These results fully proved that DHA could induce ferroptosis of HCC cells in vitro.

Lipid peroxidation has always been recognized as the central link in ferroptosis. Lipidomics studies have found that abnormal lipid metabolism is closely related to ferroptosis. Arachidonic acid and epinephrine acid in polyunsaturated fatty acids are the critical ingredients that induce ferroptosis [6, 36]. The formation of these polyunsaturated fatty acid coenzyme A derivatives, namely, oxidized phosphatidylethanolamines (ox-PEs), is the signal necessary for ferroptosis. Their formation requires the participation of a variety of lipid-metabolizing enzymes, such as acyl-CoA synthetase long-chain family member 4 (ACSL4) and lipoxygenases (LOXs). Our experimental results also showed that the accumulation of lipid peroxide MDA and 4-HNE in HCC cells was particularly obvious after DHA treatment. We speculated whether 15-LO was also involved in the ferroptosis of HCC cells induced by DHA. Our knockdown experiments showed that inhibition of 15-LO could significantly abolish the changes in ferroptosis indicators caused by DHA. However, the effect of DHA on 15-LO was not obvious at either protein or mRNA levels. Could it be that DHA does not regulate the expression of 15-LO but change its structure and function? Based on literature research, we found a scaffold protein inhibitor of the protein kinase cascade PEBP1, which was reported to bind and direct 15-LO to target polyunsaturated fatty acids (PUFAs) on the cell membrane to promote ferroptosis.

Therefore, we speculated whether DHA could induce ferroptosis in HCC cells by promoting the combination of PEBP1 and 15-LO. First, we tested the effect of DHA treatment on the combination of PEBP1 and 15-LO. We found that DHA could significantly promote the formation of PEBP1/15-LO complex via immunoprecipitation and immunofluorescence colocalization experiments. Next, our further confirmation of the role of PEBP1 indicated that interference with PEBP1 weakened the function of DHA-induced HCC cell ferroptosis.

We have confirmed that DHA-induced cell ferroptosis was resulted from inducing the formation of the PEBP1/15-LO complex based on previous results, but DHA had no remarkable effect on the expression of 15-LO. Therefore, we further wanted to know whether DHA could regulate the level of PEBP1. Interestingly, we found that DHA only regulated the expression of PEBP1 at the protein level, but not the mRNA level. Accordingly, we considered the DHA-induced upregulation of PEBP1 was due to the inhibition of protein degradation, which principally occurred in proteasome and lysosome [37]. DHA treatment could significantly prolong the half-life of PEBP1. In addition, the degradation of PEBP1 could be inhibited by proteasome inhibitor MG132, but not autophagy inhibitor CQ, implying the proteolysis of PEBP1 by the former pathway. And the polyubiquitination of PEBP1 was significantly downregulated after DHA treatment. Ubiquitin is the most frequent posttranslational modification of proteins, and more than 80% of proteins in cells are degraded by the ubiquitin-proteasome system. Ubiquitylation is widely involved in a variety of cell life processes, including cell survival and differentiation. In addition, ubiquitination also plays a crucial role in diseases, such as tumors, inflammatory disorders, and metabolic syndromes [38]. As reported by An et al. [39], USP18, a member of the deubiquitination enzyme family, plays a vital role in the development of NAFLD through inhibiting TAK1 activation and subsequently restraining the downstream JNK/NF-*κ*B signaling pathways. Therefore, the regulation of the ubiquitination pathway has been considered as a promising therapeutic strategy for those diseases [40–42].

In addition to binding with phospholipid molecules, PEBP1 can also bind to Raf-1 and inhibit its downstream signaling pathway, so it is also called RKIP, a Raf kinase inhibitor protein. Compared with normal tissue, PEBP1 is generally underexpressed in most cancers, including HCC [43]. Downregulation of PEBP1 in tumors indicates a worse clinical prognosis, which may be a beneficial biomarker to evaluate patients at high risk of recurrence after surgery. At present, the regulatory role of PEBP1 is mainly focused on its transcriptional activity [44, 45]. To our knowledge, this study is the first to report the regulation of PEBP1 expression at the protein level in HCC. Moreover, the role of PEBP1/15-LO in ferroptosis in HCC cells was also discussed for the first time in this study.

In the end, we established a subcutaneous xenograft model in nude mice to verify the antitumor effect of DHA, the ability in inducing ferroptosis of DHA, and the role of PEBP1 in these effects. All in vivo results were consistent with the cell experiment.

## 5. Conclusion

**Figure 9:**
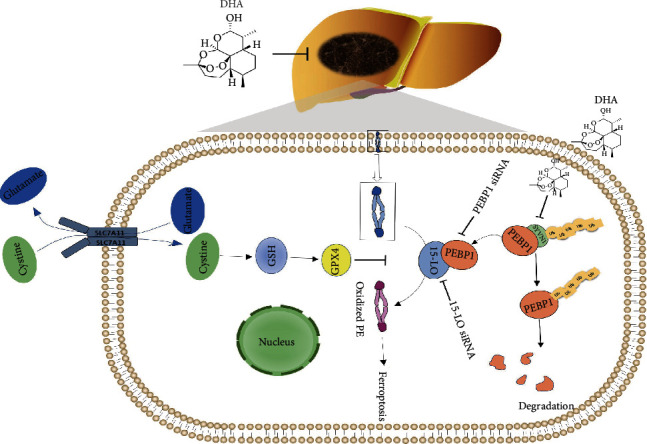
DHA inhibited HCC development by inducing the formation of PEBP1/15-LO complex to promote ferroptosis in HCC. DHA therapy can activate ferroptosis and inhibit the development of HCC. Reductive glutathione (GSH) and glutathione peroxidase 4 (GPX4) are effective in reducing lipid peroxidation and ferroptosis. PEBP1/15-LO compound may be bound up with to DHA-induced ferroptosis. 15-LO siRNA and PEBP1 siRNA were able to prevent ferroptosis induced by DHA. The upregulated PEBP1 may play a significant role in this molecular mechanism. DHA mainly inhibits the ubiquitination degradation of PEBP1 and promotes the formation of PEBP1/15-LO and induces ferroptosis.

Taken together, these results provide evidence of a mechanism by which PEBP1/15-LO is involved in the role of DHA-induced ferroptosis in HCC (Figure 9). By inhibiting the ubiquitin-proteasome pathway of PEBP1, DHA increased its protein level and bound to 15-LO, which further promoted the lipid peroxidation of HCC cell membrane to induce ferroptosis, thus exerting the anti-HCC activity of DHA.

## Figures and Tables

**Table 1:** The primer sequence.

Gene (human)	Sequence
GAPDH	
Forward	5′-GACATCAAGAAGGTGGTGAAGC-3′
Reverse	5′-TGTCATTGAGAGCAATGCCAGC-3′
GPX4	
Forward	5′-ACAAGAACGGCTGCGTGGTGAA-3′
Reverse	5′-GCCACACACTTGTGGAGCTAGA-3′
SLC7A11	
Forward	5′-ATGCAGTGGCAGTGACCTTT-3′
Reverse	5′-GGCAACAAAGATCGGAACTG-3′
PTGS2	
Forward	5′-GAATCATTCACCAGGCAAATTG-3′
Reverse	5′-TCTGTACTGCGGGTGGAACA-3′
PEBP1	
Forward	5′-GACATCAGCAGTGGCACAGT-3′
Reverse	5′-GTCACACTTTAGCGGCCTGT-3′
15-LO	
Forward	5′-TTCTGTCCCCCTGATGACTT-3′
Reverse	5′-ACGATTCCTTCCACATACCG-3′

## Data Availability

All data generated or analyzed during this study are available from the corresponding author upon request.

## Supplementary Materials

Supplementary materials Supplementary Figure 1. (a-b) Images of BALB/c nude mice tumors stripped from different groups.
