# Supplementary figures and images for: Dihydroartemisinin Induces Ferroptosis in HCC by Promoting the Formation of PEBP1/15-LO
Source: Oxid Med Cell Longev. 2021 Dec 10;2021:3456725. doi: 10.1155/2021/3456725 (PMC8683180; doi:10.1155/2021/3456725)

**
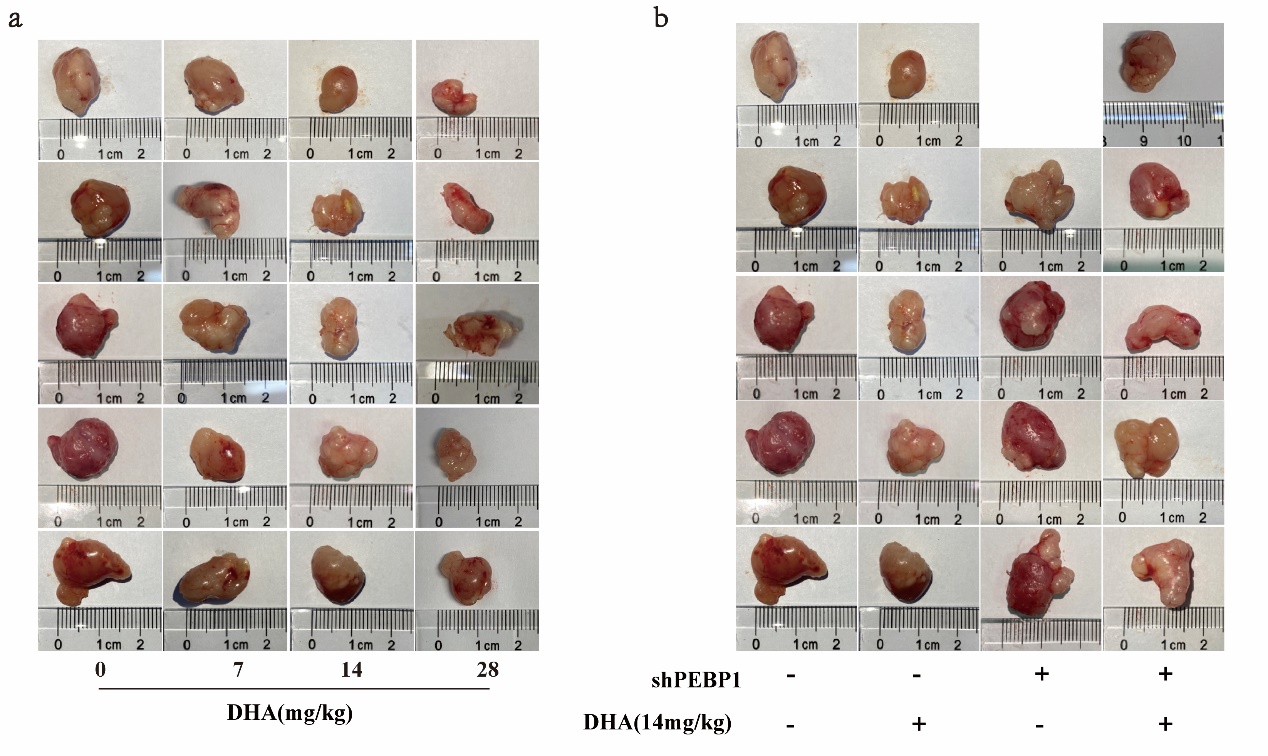
Supplementary Figure 1.** (a-b) Images of BALB/c nude mice tumors stripped from different groups.

Supplement: Supplementary materials — Supplementary Figure 1. (a-b) Images of BALB/c nude mice tumors stripped from different groups. [file 3456725.f1.docx]
